# Complete genome sequence of *Bacillus velezensis* CIMT3, isolated from maize-cultivated soil

**DOI:** 10.1128/mra.01207-24

**Published:** 2025-03-25

**Authors:** GyuDae Lee, Tino Bashizi, Jae-Ho Shin

**Affiliations:** 1 Department of Applied Biosciences, Kyungpook National University 34986 , Daegu, South Korea; 2 NGS Core Facility, Kyungpook National University 34986 , Daegu, South Korea; The University of Arizona, Tucson, Arizona, USA

**Keywords:** whole-genome sequencing, rhizosphere-inhabiting microbes

## Abstract

This report presents the complete genome sequences of *Bacillus velezensis* CIMT3, a widely recognized plant growth-promoting bacterium. The genome of this strain comprises one chromosome with 4,242,144 bp and 46% GC content.

## ANNOUNCEMENT

*Bacillus velezensis* is a gram-positive, aerobic, rod-shaped bacterium ubiquitous in diverse environments, including soil (1). It is renowned for its plant growth-promoting properties in soil ecosystems (2). While numerous *B. velezensis* strains have been isolated and characterized globally, limited genomic studies exist for strains from African agricultural ecosystems. In this study, we isolated *B. velezensis* strain CIMT3 from maize-cultivated soil in Kabare, Democratic Republic of the Congo (2°20′08″S, 28°46′59″E), by collecting rhizosphere soil that was tightly adhered to 1-month-old maize roots after carefully removing the bulk soil. Briefly, 1.0 ± 0.1 g of homogenized soil sample was suspended in 9.0 mL of sterile 0.85% (wt/vol) NaCl solution. Subsequently, 100 µL of this suspension was spread plated onto potato dextrose agar (Difco, USA) plates. A single colony of the isolated strain was then cultured in potato dextrose broth medium at 30°C for 18 h with agitation at 200 rpm.

Genomic DNA for whole-genome sequencing was extracted using the Wizard Genomic DNA Purification Kit (Promega, USA) according to the manufacturer’s instructions. Prior to whole-genome sequencing, taxonomic identification was performed using 16S rRNA gene amplification. PCR was conducted using universal bacterial primers 27F (5′-AGAGTTTGATCMTGGCTCAG-3′) and 1492R (5′-TACGGYTACCTTGTTACGACTT-3′). PCR conditions were as follows: initial denaturation at 94°C for 5 min, followed by 30 cycles of denaturation at 94°C for 30 s, annealing at 55°C for 30 s, and extension at 72°C for 1 min, with a final extension at 72°C for 10 min. The amplified PCR products were sequenced using Sanger sequencing at Macrogen Inc. (Seoul, South Korea), and the sequence was submitted to the National Center for Biotechnology Information (NCBI) database under accession number PQ804475. The quantity and quality of extracted DNA were assessed using a Qubit 3.0 Fluorometer (Invitrogen, USA) and a NanoDrop One Spectrophotometer (Thermo Fisher Scientific, USA), respectively. DNA samples that passed quality control criteria based on Qubit and NanoDrop measurements were selected for Oxford Nanopore Technologies (ONT) sequencing without prior shearing. Library preparation was performed using the Ligation Sequencing Kit (SQK-LSK109, ONT, Oxford, UK) without prior shearing, and sequencing was conducted on a MinION device equipped with a FLO-MIN111 (R10.3) flow cell (ONT). Long-read sequencing was performed in the KNU NGS Core facility, Daegu, South Korea. Basecalling of the sequencing data was performed using Guppy software (version 4.4.1) high accuracy model, yielding a total of 132,912 reads with an aggregate length of 531,627,083 base pairs (bp) (3).

Raw sequencing data underwent initial quality control using Filtlong version 0.2.1 with default parameters to remove the bottom 5% quality reads (4). Subsequently, *de novo* genome assembly was performed using the high-quality long reads obtained from the ONT MinION device. The draft genome was assembled using Flye version 2.9.1 with the following parameters: “--nano-raw --genome-size 4 m,” while other parameters were set to default (5). This assembly process included an inherent error correction step. The final polishing of the assembled contig was carried out using Medaka version 1.7.2 with high-quality ONT reads (6).

**Fig 1:**
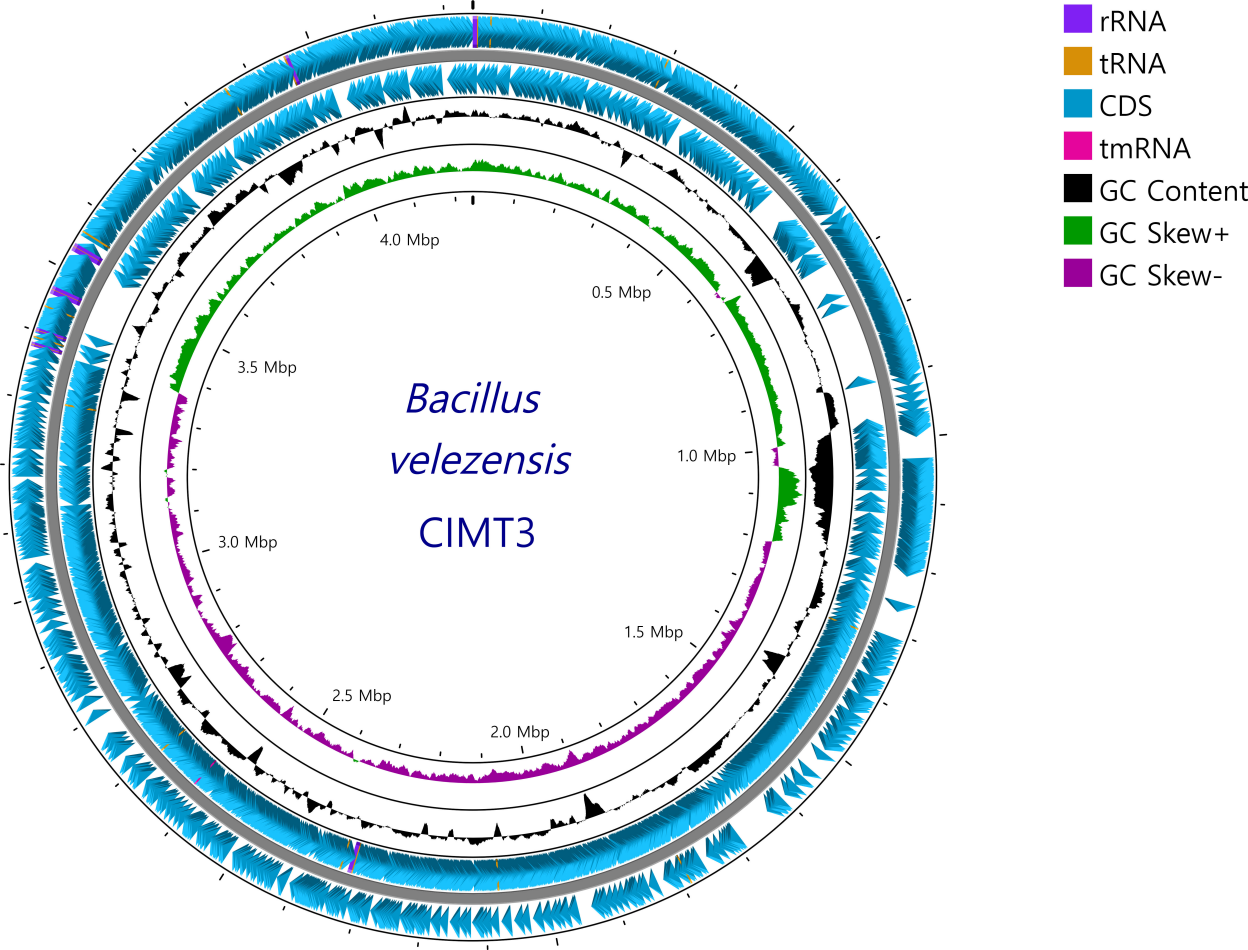
Circular genome map of *B. velezensis* CIMT3. From outside to inside: forward and reverse strands showing coding DNA sequences (CDS, blue), rRNA (purple), tRNA (gold), and tmRNA (pink) genes; GC content (black); GC skew (green: positive and purple: negative); and genome size markers at 0.5 Mbp intervals (total genome size: 4.0 Mbp).

Following the polishing step, the final genome assembly of *B. velezensis* CIMT3 yielded a total size of 4,242,144 bp with 98× coverage, comprising a single circular chromosome. The circularity of the assembly was confirmed through dot plot analysis using Gepard (7). Genome visualization was performed using Proksee (Fig. 1) (8). Functional gene identification and annotation were carried out using the NCBI Prokaryotic Genome Annotation Pipeline (9). The annotation process predicted a total of 4,256 genes, including 4,042 protein-coding genes and 118 RNA genes. Among the RNA genes, 27 were identified as rRNA genes and 86 as tRNA genes. Additionally, 96 pseudogenes were detected in the genome.

## Data Availability

The assembly data of *B. velezensis* CIMT3 have been submitted to NCBI GenBank and have been deposited under the accession number CP169570.1, BioProject number PRJNA1153383, and BioSample number SAMN43392604. Additionally, raw reads were available under SRA accession number SRR31209720, and the 16S rRNA gene sequence is available under accession number PQ804475.
